# The Assessment of the Reduction Algorithm in the Treatment for “Logsplitter” Injury

**DOI:** 10.1155/2020/4139028

**Published:** 2020-03-10

**Authors:** Kangquan Shou, Richa Adhikary, Liang Zou, Hao Yao, Huarui Yang, Krishna Adhikary, Yi Yang, Tongzhu Bao

**Affiliations:** ^1^Department of Orthopedic, The First College of Clinical Medical Science, China Three Gorges University & Yichang Central People's Hospital, Yichang 443002, China; ^2^Helping Hands Community Hospital, Chabahil, Kathmandu, Nepal

## Abstract

As a rare and exceptional injury with significant syndesmotic disruption, the outcome of Logsplitter injury remains poor and unfavorable. In this study, we retrospectively investigated the relationship between the intraoperative reduction quality and the prognosis such as the posttraumatic osteoarthritis to help surgeons achieve better functional outcomes for this high-energy transsyndesmotic ankle fracture dislocation. From January 2015 to February 2019, 31 patients (average 37.6 ± 9.4 years with 19 male and 12 female) diagnosed with the Logsplitter injury were treated by ORIF procedure and enrolled in our study. Particularly, nine vital radiographic parameters including medial clear space, talocrural angle, superior clear space, tibiofibular clear space, tibiofibular overlap, talar tilt, coin sign, tibial medial malleolus angle, and fibular lateral malleolus angle were measured from a postoperative film (AP and mortise view). Next, we compared the clinical outcome by using range of ankle motion, AOFAS scores, Burwell-Charnley score system, and Kellergen-Lawrence criteria from the patients who obtained the intraoperative anatomical reduction with those who failed. Our results showed that AOFAS score with all the patients was 79.33 ± 5.82 at the final follow-up. 14 (45.1%) of 31 patients were observed with radiographic posttraumatic arthritis of the ankle joint with an average Kellgren-Lawrence score of 1.75 ± 1.6 at final follow-up. Most importantly, our results proved that there were significant differences between the patients eligible for anatomical reduction quality with those who failed with regard to OA rate (33.3% vs. 85.7%, *P* = 0.003) and AOFAS scores (75.33 ± 6.53 vs. 66.89 ± 4.28, *P* = 0.037) at the final follow-up. Furthermore, the functional outcome after the operation showed an increased range of motion of the ankle joint of the patients obtained anatomical reduction compared with those who failed (*P* < 0.05). In this study, the significant discrepancy with regard to the functional outcomes was observed between the acceptable and unacceptable radiographic parameters, indicating that the quality of intraoperative reduction is scientifically significant and thus can be utilized as the major factor to predict the clinical outcomes for Logsplitter injuries. Moreover, this reduction algorithm arising from our study can also be applied to other ankle fractures and dislocation involving syndesmotic complex.

## 1. Introduction

Ankle fracture is a common injury with potentially significant morbidity, accounting for 9% of all fractures [1]. Distal tibiofibular syndesmosis disruption is considered one of the most severe injury associated with ankle joint. Currently, a special injury, “Logsplitter” injury, first reported by Bible et al. [2], draws a lot of attention for foot and ankle surgeons. In regard to this injury, the patients may suffer from the high-energy trauma with the talus wedging vertically into the distal tibiofibular joint, thus giving rise to the extraordinary injury featured as the syndesmotic displacement. It is also featured as the fracture involving the tibial plafond and distal fibula. This injury was named as “Logsplitter” as the injury process is characterized as the talus punching into the inferior tibiofibular joint just like a logsplitter wedge splitting into the firewood by a sledgehammer. It represents an exceptional pattern of high-energy fractures comprising significant syndesmotic disruption, potential soft tissue compromise, and possible associated tibial plafond injuries. Previously, it was reported with a very bad prognosis with 70% radiographic evidence of posttraumatic ankle arthritis, 17% infection, 17% nonunion rate, and very low functional scores (AOFAS = 67.0 ± 26.8) [3].

Given the fact that “Logsplitter” injury was first described only a few years back, there is a high possibility that these injuries have not been fully understood yet, leading to the lack of its optimal treatment strategies. In other words, the baseline still remains that we do not have an approved treatment protocol for Logsplitter injuries in the current scenario. Previously, Wang et al. [4] concluded that the patients with atypical injury showed a better range of motion of the ankle joint and low incidence rate of posttraumatic ankle arthritis compared with those typical ones, indicating that the postoperative outcome was strongly affected by the injury pattern at the beginning stage of injury. However, we believe that most of these cases are treated according to the traditional ankle fractures or pilon fractures, which are completely different from the “Logsplitter” injury, thus resulting in poor prognosis. Notably, AO recommends that whenever and however the displacement is, ankle injuries are considered unstable, and only by reliable internal fixation accurate anatomic reduction can be ensured. Particularly, the “Logsplitter” injury is an exceptional pattern of transsyndesmotic ankle fracture dislocations, representing the severe damage of the anatomical structure of the ankle joint, which means we need to figure out how to achieve the anatomical reconstruction of the ankle joint.

In this study, we tried to focus on the intraoperative reduction quality and evaluated some key parameters to help orthopedic surgeons achieve better functional outcomes with this injury in the future. We hypothesis that good intraoperative reduction quality in the surgery is the key factor to achieve better functional outcomes instead of the injury pattern. From this study, we conclude that if Logsplitter injuries are intraoperatively reduced keeping the radiographic parameters in mind, there may be high chances of better functional outcomes in the future.

## 2. Materials and Methods

### 2.1. Patients

This is a retrospective cohort study. From January 2015 to February 2019, a total of 31 patients admitted to the First Clinical Medical College of Three Gorges University and Yichang Central People's Hospital were retrospectively analyzed on the basis of the inclusion criteria. The inclusion criteria consisted of (1) AO/OTA classified 44A, 44B, and 44C fractures; (2) radiographic films consistent with the diagnostic criteria of Logsplitter injury; (3) an obvious vertical axial force and/or combined with a rotational force, giving rise to the ankle joint dislocation; (4) syndesmosis disruption; and (5) axial displacement of talus above the tibial plafond. The exclusion criteria included (1) patients with severe systemic diseases who cannot undergo surgery (such as shock, damage to vital organs, poorly controlled diabetes mellitus, and serious cardiovascular and cerebrovascular diseases), (2) poor blood coagulation profile, and (3) preexisting osteoarthritis. The Logsplitter injury in this study was confirmed according to the previous reports [2]. All the procedures were approved by the Ethics Committee of our hospital. A written informed consent was completed from each patient after explaining the details of the study.

The mean age for the patients enrolled in our study was 37.6 ± 9.4 (21–59) years with 19 male and 12 female. The most frequent mechanisms of injury included falls from height (16 cases), followed by traffic accidents (10 cases) and falling down in 5 cases, respectively. By using AO/OTA classification, the fractures were categorized as 44A (*n* = 1), 44B (*n* = 5), and 44C (*n* = 25). There were 17 patients with closed injuries whereas 14 patients with open fractures (most of the wound located at the medial side accompanied with the medial malleolus extruding via the open wound).

### 2.2. Operative Techniques

With regard to the patients with closed fractures, manual reduction and continuous calcaneal traction were first performed, and the open reduction and internal fixation (ORIF) procedure were performed until the appearance of the “wrinkle sign,” indicating soft tissue swelling subsidence. For those patients with open fractures, initial debridement was performed combined with the limited internal fixation by the Kirschner wire or external fixation (supporter) as the temporary stabilization for the ankle joint, followed by vacuum-assisted closure (VAC) as the cover of wound. After several times of debridement and change of VAC, the second-stage surgery (terminal surgery) was conducted until skin conditions were guaranteed. Briefly, the patient was placed in supine position on the operating table and were given combined spinal-epidural anesthesia or general anesthesia. The medial “J” incision and posterolateral straight incision were made to expose the fracture fragments, and then the reduction of displaced fragments were completed. Then, the distal fibular fractures were fixed by using the LCP plates and the medial malleolus fracture was fixed using two cannulated screws or the Kirschner wire. Additionally, the “Volkmann” fragments or the “Chaput” fragments were fixed by using the plates or the cannulated screws. After the “Hook” tests were performed to assess the stability of the inferior syndesmotic complex, one or two syndesmotic screws were used to fix the syndesmosis joint. Next, wounds were irrigated thoroughly. Finally, the wounds were sutured layer by layer using absorbable sutures. Dressing followed by a splint with extra padding under the heel is applied for immobilization.

### 2.3. Postoperative Management

Early functional exercise such as dorsal expansion and plantar flexion of the ankle joint was encouraged after 2-3 weeks of immobilization by using the cast or splint. Partial weight bearing was allowed 6 weeks after the surgery under the protection of the personalized functional brace. Patients were followed up for the wound, bone union, range of motion, ankle joint function, and postoperative traumatic arthritis at postoperative 3 months, 6 months, 12 months, and 2 years. In the last follow-up, patients were assessed with a range of ankle motion (plantar flexion, dorsal expansion, and eversion and inversion for ankle joint), AOFAS scores [5], Burwell-Charnley score system, and Kellergen-Lawrence criteria [2] for traumatic arthritis to assess the functional outcome.

### 2.4. Radiographic Evaluation

All the radiographic parameters were measured in our hospital using the computer software by two independent radiologists in a one blind manner, who were trained in how to take radiographs in this study. Three radiographic views including AP (anteroposterior) view, mortise view, and lateral view X-ray scans were performed in all patients.

### 2.5. Measurements

We utilized several parameters that are considered the most reliable characteristics related to the normal ankle joint [6–8]:

1. Medial clear space (MCS): the medial clear space lies between the lateral border of the medial malleolus and the medial border of the talus. (<4 mm is normal.)
2. Talocrural angle (TCA): talocrural angle was measured as the angle created from a line perpendicular to the distal tibial articular surface and a line connecting the tip of medial and lateral malleolus. (<78° is normal)
3. Superior clear space (SCS): SCS was measured as the distance from the inferior border of the articular surface of the distal tibia to the talar dome at the midpoint of the distal tibial articular surface.(<4 mm is normal, equal with MCS is optimal)
4. Tibiofibular clear space (TFCS): tibiofibular clear space was measured from the medial border of the distal fibular to the medial border of the incisura fibularis at 1 cm proximal to the distal tibial articular surface. (<4 mm is normal)
5. Tibiofibular overlap (TFOL): tibiofibular overlap was measured as the distance of overlapping from the medial border of the lateral malleolus to the lateral border of the anterior tibial tubercle at 1 cm proximal to the distal tibial articular surface. (>6 mm in AP view or >1 mm in mortise view)
6. Talar tilt (TT): a line drawn parallel to the articular surface of the distal tibia should be parallel to a line drawn parallel to the articular surface of the talus
7. Coin sign (CS): unbroken curve between the lateral talar articular surface and recess of the distal fibula
8. Tibial medial malleolus angle (TMMA): this is the angle measured between a parallel line drawn through the articular surface of the talus and line through the tip of the medial malleolus. (52° ± 3 as the standard)
9. Fibular lateral malleolus angle (FLMA): this is the angle measured between a parallel line drawn through the articular surface of the talus and line through the tip of the lateral malleolus. (53° ± 3 as the standard)

### 2.6. Statistical Analysis

Statistical analysis was performed by using SPSS 20.0 software. Different ranges of motion measured for each time point and for those subgroups with anatomical reduction or not were compared by using Student's *t* tests. The AOFAS score and K-L score were evaluated by Student's *t* tests as well. The incident rate of osteoarthritis was compared by using Fisher's exact test in each small proportion analysis. A significance level of *P* < 0.05 was set as significant.

## 3. Results

Ultimately, mean follow-up was 22.9 ± 3.3 (13–26) months. For all the patients, AOFAS score at the final follow-up was 79.33 ± 5.82 (Table 1). Additionally, 14 (45.1%) of 31 patients were observed of radiographic posttraumatic arthritis of the ankle joint with an average Kellgren-Lawrence score of 1.75 ± 1.6 at final follow-up.

With regard to the AOFAS scores, there was a significant improvement at 3 months follow-up compared with preoperative observation (^∗^*P* < 0.05). Notably, there was a significant improvement by comparing the follow-up between 3 and 24 months, but no significant improvement was observed between 3 months and 6 months follow-up, indicating the accelerated functional recovery at the late stage after operation. The *P* value for all the range of motion of comparison between 3 months and 24 months follow-up was <0.05, suggesting that there was improvement for the movement of the ankle joint along with increasing time. The Burwell-Charnley scoring system, which is considered the classic evaluation system for the reduction quality of the operative procedure, showed that 8 poor reduction occurred at 24 months final follow-up. In addition, average 1.75 ± 1.6 was observed at the final follow-up for all patients (Table 1).

To establish the relationship between the quality of intraoperative reduction and functional outcome, we analyzed 9 radiographic parameters, dividing the cases with acceptable radiographic parameters with those which did not fulfill the acceptable measurements. The *P* values for the range of motion was statistically significant (*P* < 0.05) in all the parameters except for TMMA (*P* > 0.05), suggesting that TMMA was probably not the sensitive parameter for predicting the OA after surgery. Most importantly, the AOFAS score was scientifically better (*P* < 0.05) in all the subgroups, those eligible for anatomical reduction quality compared with those who failed (Table 2).

**Figure 1:**
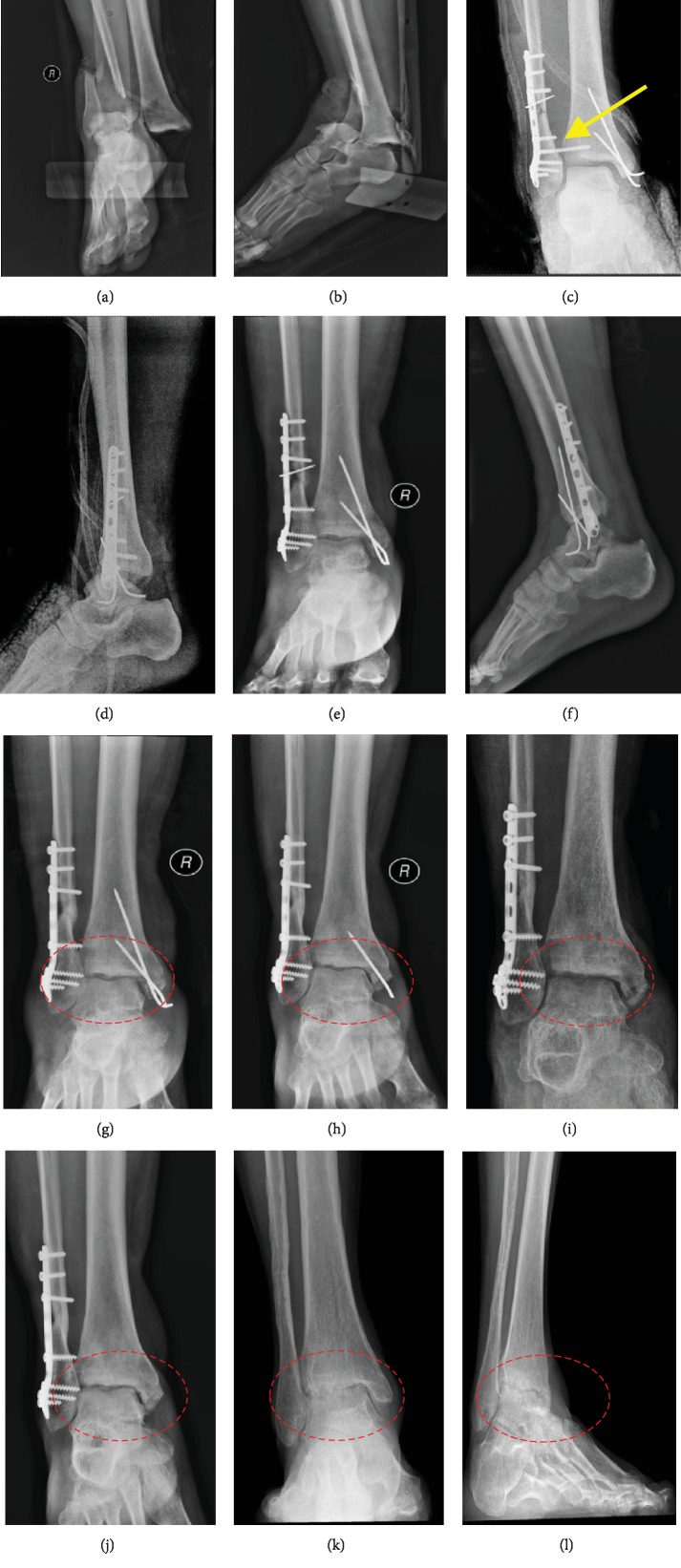
Preoperation (a, b) and postoperation radiographs after first reduction and debridement (c, d) from a 37-year-old man who suffered from falling from a height, showing the malreduction of the lower tibiofibular joint (yellow arrow). Thus, we performed revised surgery (e, f) to obtain acceptable anatomy of TFOL and TFCS. However, gradual degeneration of the tibiotalar joint and deterioration of the joint surface were observed along with the different follow-up (red circle with dot line) (g–j). Finally, significant posttraumatic osteoarthritis occurred at the ankle joint at the last follow-up (k, l).

Additionally, we assessed whether there was a unique relationship between the reduction quality and the incident rate of postoperative osteoarthritis. Our results showed that there was a significant difference between these two groups with regard to OA rate (33.3% vs. 85.7%, *P* = 0.003) and AOFAS scores (75.33 ± 6.53 vs. 66.89 ± 4.28, *P* = 0.037) at the final follow-up, illustrating the lower posttraumatic osteoarthritis rate and better outcome if we obtained anatomical reduction according to the normal alignment of the ankle joint. Furthermore, the functional outcome after the operation showed an increased range of motion of the ankle joint of the patients who obtained anatomical reduction compared with those who failed (*P* < 0.05) (Table 3). The typical case with unfavorable intraoperative reduction was shown in Figure 1, indicating that the obvious posttraumatic osteoarthritis occurred at the ankle joint when the follow-up was observed.

## 4. Discussion

Since first reported by Bible et al. [2], Logsplitter injury is featured as an exceptional pattern fracture caused by high energy with significant syndesmotic disruption and potential soft tissue compromise. Only a few studies focused on this unusual injury; however, unfavorable prognosis accompanied by a high incident rate of posttraumatic ankle arthritis has been reported [4]. The authors concluded that the typical type of Logsplitter injury showed worse outcome and functional prognosis compared with atypical type, indicating that the initial injury status would be the predicted factor for long-term prognosis. However, we believe that intraoperative reduction is the key factor for better prognosis. Therefore, in this study, we focused on the intraoperative reduction quality for Logsplitter injury and tried to investigate several vital anatomical parameters to help orthopedic surgeons achieve better functional outcomes. We concluded that anatomical reduction is essential and indispensable for a complicated dislocation and fracture occurring at the ankle joint, especially for Logsplitter injury. Even if the patients with Logsplitter injury suffer from severe damage upon the distal tibiofibular joint, surgeons can still have the chance to obtain favorable clinical outcome once anatomical reduction is accomplished. Our findings may pave a new way for foot and ankle surgeons to make correct decisions and keep the anatomical reduction in mind during the operation for better clinical outcome.

If the detailed mechanism of Logsplitter injury is carefully examined, we will find out that the ankle joint surface and cartilage is easily involved during the high-energy axial violence striking, leading to the articular cartilage injury or collapse fracture. AO believes that all unstable intra-articular fractures require operation to ensure anatomical reduction as it is closely related to therapeutic effect and prognosis [9]. It has been proved that the instability of the ankle joint will give rise to the articular stress changes, thus accelerating the degeneration of the cartilage, which is considered the main pathological factor for long-term traumatic osteoarthritis [10]. Since the salvage surgery such as ankle replacement or ankle joint fusion is the only option for those posttraumatic osteoarthritis with severe comminuted damage, it is worthy to obtain the anatomical reduction when the first surgery is performed to prevent potential post-traumatic osteoarthritis [11].

During the injury process of Logsplitter injury, when the talus moves outward, the separation injury of the lower tibiofibular joint would be inevitable [12]. Kennedy et al. [13] also found that poor reduction of the lower tibiofibular joint could lead to abnormal biomechanics of the ankle, leading to a significant increase in the incidence of postoperative ankle pain and long-term arthritis. Leeds and Ehrlich [14] reported that the incidence of long-term arthritis would be significantly increased if the gap between the medial fibula wall and the lateral tibial posterior malleolus wall (tibiofibular clear space (TFCS)) on the posterior-anterior film of the ankle was more than 5 mm or 2 mm compared with the normal side. Additionally, Mulligan [7] concluded that the medial clear space (MCS) was supposed to be less than 4 mm, otherwise the disruption of syndesmotic complex would be highly suspected. Amendola et al. [15] reported that tibiofibular overlap (TFOL, measured at 1 cm proximal to the distal tibial articular surface) should be larger than 6 mm in AP view or 1 mm in mortise view. In our study, we introduced these three vital parameters to evaluate the intraoperative reduction quality especially the reconstruction of syndesmotic complex. Our findings demonstrated that if the perfect results including MCS, TFOL, and TFCS were obtained during the operation, the better clinical outcome and decreased OA rate would be achieved (AOFAS score: 74.91 ± 10.57, 70.66 ± 12.53, and 77.92 ± 13.63; posttraumatic osteoarthritis rate: 29.2%, 29.2%, and 36%, respectively. *P* < 0.05, compared with those patients who failed to obtain anatomical reduction. *P* < 0.05).

More importantly, the axial, transverse slip, and rotation of the fibula and lower tibiofibular joints are not consistent, suggesting that the shortening and rotation of the fibula needs to be further carefully assessed. Previously, several studies proved that the anatomical reduction and fixation of the fibula are the key factors for the prognosis of ankle fracture and dislocation [16, 17]. The common errors in fibula reduction are shortening, displacement, and rotation. Poor reduction of the fibula can give rise to abnormal alignment and then result in significant changes in the biomechanics of the ankle joint. Subsequently, the pain around the ankle joints will emerge when functional exercise and weight-bearing activities begin. Thus, to prevent malalignment of the fibular such as shortening and rotation, reliable reduction and anatomical realignment of the fibular need to be achieved and any shortening or displacement of the fibula should be corrected. Rungprai [18] measured the angle from a line perpendicular to the distal tibial articular surface and a line connecting the tip of the medial and lateral malleolus and named it as the talocrural angle (TCA), reflecting the normal length of the fibula what the surgeons need to keep during the surgery. Webber and Simpson [6] determined that there was an unbroken curve existing between the lateral talar articular surface and recess of the distal fibula, which was also known as “coin sign” and considered the most effective parameter to assess the reductive quality of the fibula in an ankle joint surgery [19]. In our study, we measured the TCA, coin sign, and fibular lateral malleolus angle (FLMA) to evaluate the reduction quality of the fibula. Our findings illustrated that if the normal length of the fibula and anatomical relationship around the distal fibula was restored, the functional outcome would be better and the incident rate of osteoarthritis would be avoided.

Furthermore, we also tried to establish a new reduction algorithm by assembling all these parameters to describe the relationship between the intraoperative quality and the clinical outcome. Our findings indicated that if we obtained the anatomical reduction following all these reduction algorithms in the treatment for “Logsplitter” injury, the posttraumatic osteoarthritis rate would decrease from 85.7% to 33.3% (*P* < 0.01). Meanwhile, the better clinical prognosis will also be achieved (75.33 ± 6.53 vs. 66.89 ± 4.28, *P* < 0.05) and lower Kellergen-Lawrence will be observed as well (0.62 vs. 1.83, *P* < 0.01).

Last but not the least, since Logsplitter injuries falls in a similar pattern with other complicated fracture and dislocation of the ankle joint such as “Dupuytren fracture” and “Bosworth fracture,” our results can also be applied for other injuries to emphasize the importance of intraoperative reduction quality. Our findings demonstrated that the radiographic parameters enrolled in our study must be kept in mind for foot and ankle surgeons when the operation is performed. Therefore, a standard treatment protocol needs to be created for injuries involving the lower tibiofibular syndesmosis in the future.

There are some weaknesses in the study. First, since Logsplitter injuries are clinically rare, the number of the cases enrolled in our study is limited. Although we assume that our results could provide several vital information and guide our future work for those complicated fracture and dislocation around the ankle joint, we are still planning to enroll more patients into our study in the next stage. Second, the follow-up duration needs to be extended for more solid evidence of posttraumatic osteoarthritis.

## 5. Conclusion

Our study reported that the quality of intraoperative reduction instead of the cause and mechanism of the injury may play a major role in the functional outcome of Logsplitter injuries. The significant discrepancy with regard to the functional outcomes was observed between the acceptable and unacceptable radiographic parameters, indicating that the quality of intraoperative reduction is scientifically significant and thus can be utilized as the major factor to predict the clinical outcomes for Logsplitter injuries. Moreover, this reduction algorithm arising from our study can also be applied to other ankle fractures and dislocation involving syndesmotic complex.

## Figures and Tables

**Table 1:** Postoperative outcomes of patients with Logsplitter injury.

	Range of motion				Burwell-Charnley score (good/fair/poor (*n*))	AOFAS score	Kellergen-Lawrence grading scale
	Plantarflexion	Dorsal expansion	Eversion	Inversion			
Preoperative	—	—	—	—	—	41.22 ± 8.55	—
F/U at 3 months	18.57 ± 2.91	8.33 ± 2.59	8.96 ± 1.87	19.83 ± 3.74	16/9/6	60.63 ± 5.28^∗^	—
F/U at 6 months	21.19 ± 3.81	10.86 ± 6.25	10.49 ± 2.62	21.62 ± 3.16	16/9/6	64.53 ± 9.20^**△**^	—
F/U at 12 months	23.27 ± 4.21	11.38 ± 3.19	10.58 ± 4.27	22.94 ± 4.15	16/9/6	73.53 ± 7.62	—
F/U at 24 months	25.88 ± 3.58^∗^	12.91 ± 4.08^∗^	11.18 ± 3.82^∗^	24.56 ± 5.02^∗^	16/7/8	79.33 ± 5.82^#^	1.75 ± 1.6

Data were presented as average ± SD. AOFAS score: compared with preoperative, ^∗^*P* < 0.05; compared with 3 months follow-up, ^△^*P* > 0.05, ^#^*P* < 0.01. Range of motion: compared with 3 months follow-up, ^∗^*P* < 0.05.

**Table 2:** Relationship between radiographic parameter and functional outcome.

Parameter	Definition	OA rate	Range of movement (°)				AOFAS score	K-L score
			Plantar flexion	Dorsal expansion	Eversion	Inversion		
TT	Parallel	8/25 (32%)	26.33 ± 4.08	13.22 ± 5.17	12.67 ± 3.91	24.11 ± 4.22	79.69 ± 9.72	0.58
	Not parallel	6/6 (100%)	21.46 ± 6.11	9.55 ± 3.21	8.31 ± 4.21	20.42 ± 2.51	67.62 ± 8.44	2.16
*P* value		0.018	0.039	0.041	0.033	0.046	0.035	0.039
TCA	<78°	6/22 (27.3%)	29.72 ± 2.33	14.71 ± 6.42	13.21 ± 6.42	20.51 ± 3.13	77.92 ± 13.63	0.87
	>78°	8/9 (88.9%)	24.08 ± 1.52	10.15 ± 4.13	10.41 ± 2.87	17.44 ± 3.12	70.43 ± 9.57	2.33
*P* value		0.024	0.042	0.051	0.043	0.032	0.044	0.041
SCS	<4 mm	10/26 (38.5%)	26.55 ± 4.37	15.07 ± 4.27	14.62 ± 5.28	21.42 ± 2.87	76.53 ± 9.33	0.94
	>4 mm	4/5 (80%)	20.36 ± 3.11	11.57 ± 3.92	10.92 ± 3.16	16.44 ± 2.92	69.32 ± 9.83	2.59
*P* value		0.031	0.047	0.043	0.039	0.032	0.038	0.039
TFCS	<4 mm	9/25 (36%)	28.66 ± 3.52	12.53 ± 4.37	13.14 ± 4.12	25.66 ± 2.87	77.92 ± 13.63	0.83
	>4 mm	5/6 (83.3%)	22.35 ± 4.21	8.76 ± 5.42	9.14 ± 5.37	19.82 ± 3.45	70.43 ± 9.57	2.14
*P* value		0.032	0.045	0.041	0.041	0.042	0.047	0.044
TFOL	>6 mm	7/24 (29.2%)	30.05 ± 2.57	14.56 ± 5.23	12.56 ± 4.85	23.62 ± 3.13	70.66 ± 12.53	0.47
	<6 mm	7/7 (100%)	27.52 ± 12.31	9.42 ± 3.11	9.21 ± 3.13	19.42 ± 4.53	67.31 ± 8.27	2.86
*P* value		0.017	0.051	0.047	0.046	0.044	0.046	0.041
MCS	<4 mm	7/24 (29.2%)	29.71 ± 5.37	14.47 ± 5.62	11.59 ± 3.62	27.47 ± 2.91	74.91 ± 10.57	0.51
	>4 mm	7/7 (100%)	20.22 ± 4.21	9.44 ± 3.19	8.27 ± 4.95	20.04 ± 4.17	67.43 ± 8.29	2.75
*P* value		0.019	0.046	0.041	0.042	0.041	0.042	0.042
Coin sign	Yes	8/24 (33.3%)	32.68 ± 4.73	15.82 ± 6.82	13.11 ± 6.14	24.82 ± 5.31	76.21 ± 11.97	0.83
	No	6/7 (85.7%)	25.82 ± 9.77	10.56 ± 4.28	8.19 ± 4.15	17.33 ± 5.24	65.22 ± 7.82	2.04
*P* value		0.028	0.043	0.046	0.041	0.040	0.038	0.045
TMMA	Standard	10/22 (45.4%)	25.37 ± 4.81	11.26 ± 2.81	11.29 ± 3.37	26.39 ± 3.86	71.38 ± 11.59	1.32
	Not standard	4/9 (44.4%)	22.16 ± 3.87	9.82 ± 4.28	10.19 ± 4.16	24.57 ± 3.48	62.08 ± 8.29	1.86
*P* value		0.877	0.056	0.053	0.055	0.051	0.045	0.061
FLM	Standard	8/23 (34.7%)	31.58 ± 3.91	17.82 ± 3.69	16.99 ± 3.69	28.58 ± 3.28	78.39 ± 11.57	0.89
	Not standard	6/8 (75%)	25.27 ± 5.31	10.65 ± 4.25	9.58 ± 4.12	16.28 ± 4.48	65.22 ± 3.77	2.44
*P* value		0.038	0.042	0.041	0.044	0.036	0.038	0.037

Data were presented as average ± SD or percentage (absolute number). *t* test was used to analyze continuous variables, and Fisher's exact test was used to assess the categorical variables. A *P* value of ≤ 0.05 was considered statistically significant.

**Table 3:** Relationship between reduction quality and functional outcome.

Reduction quality	n	OA rate	Range of movement (°)				AOFAS score	K-L score
			Plantar flexion	Dorsal expansion	Eversion	Inversion		
Anatomical reduction	24	8/24 (33.3%)	27.52 ± 3.72	12.47 ± 4.28	11.51 ± 2.84	22.63 ± 2.85	75.33 ± 6.53	0.62
Nonanatomical reduction	7	6/7 (85.7%)	20.24 ± 2.89	9.04 ± 4.18	8.93 ± 3.91	17.68 ± 3.37	66.89 ± 4.28	1.83
*P* value	—	0.003	0.035	0.042	0.041	0.039	0.037	0.041

Data were presented as average ± SD or percentage (absolute number). *t* test was used to analyze continuous variables, and Fisher's exact test was used to assess the categorical variables. A *P* value of ≤ 0.05 was considered statistically significant.

## Data Availability

This is an open access article distributed under the Creative Commons Attribution License, which permits unrestricted use, distribution, and reproduction in any medium, provided the original work is properly cited.
